# Exploring reporting quality of systematic reviews and Meta-analyses on nursing interventions in patients with Alzheimer’s disease before and after PRISMA introduction

**DOI:** 10.1186/s12874-018-0622-7

**Published:** 2018-11-29

**Authors:** Xiao Sun, Xiaobin Zhou, Yan Yu, Haihua Liu

**Affiliations:** 1 Department of Epidemiology and Health Statistics, School of Public Health, Qingdao University, No. 38, Dengzhou Road, Qingdao, 266021 Shandong China; 2 Department of Cardiovascular, Second Hospital, Shanxi Medical University, No.56 South Xinjian Road, Taiyuan, Shanxi China

**Keywords:** Reporting quality, Alzheimer’s disease, Nursing interventions, Systematic review

## Abstract

**Background:**

Systematic reviews (SRs) and meta-analyses (MAs) are distillation of current best available evidence, but are potentially prone to bias. The bias of SRs and MAs comes from sampling bias, selection bias and within study bias. So, their reporting quality is especially important as it may directly influence their utility for clinicians, nurses, patients and policy makers. The SRs and MAs on nursing interventions in patients with Alzheimer’s disease (AD) have been increasingly published over the past decade, but the reporting quality of article has not been evaluated after the introduction of Preferred Reporting Items for Systematic Reviews and Meta Analyses (PRISMA) Statement.

**Methods:**

According to the inclusion and exclusion criteria, we searched the databases including PubMed, EMBASE and The Cochrane Library from inception through October 16th 2018. Two reviewers independently selected articles and extracted data. The PRISMA checklist was adopted to evaluate reporting quality. Comparisons were made between studies published before (2001–2009) and after (2011–2018) its introduction.

**Results:**

A total of 77 eligible articles, 18 (23.4%) were published before the PRISMA Statement and 59 (76.6%) were published afterwards. There was higher score after publication of the PRISMA Statement than before (20.83 ± 3.78 vs 17.11 ± 4.56, *P* <  0.05). There was an improvement in the following items after the PRISMA statement was released (*P* <  0.05): title (item 1, 50.0% vs 74.6%, OR = 3.10, 95CI%: 1.00–9.61), search (item8, 27.8% vs 57.6%,OR = 3.25, 95CI%: 1.14–9.28), study selection (item 9, 44.4% vs 81.4%,OR = 6.28, 95CI%: 1.93–20.37), Data collection process (item 10, 50.0% vs 76.3%,OR = 3.45, 95CI%:1.10–10.84), risk of bias in individual studies (item 12, 50.0% vs 83.1%, OR = 5.78, 95CI%:1.71–19.52), risk of bias across studies (item15, 5.6% vs 28.8%,OR = 3.60, 95CI%:1.04–12.43), study characteristics (item 18, 77.8% vs 98.3%, OR = 28.13, 95CI%:3.35-236.19), risk of bias with studies (item 19, 50.0% vs 83.1%, OR = 5.78, 95CI%:1.71-19.52), results in individual studies (item 20, 72.2% vs 94.9%, OR = 11.09, 95CI%:1.99–61.82), conclusions (item 26, 77.8% vs 98.3%, OR = 28.13, 95CI%:3.35–236.19). After controlling for the confounding factors, there were higher PRISMA score for systematic reviews including meta-analyses, protocol or registration, can’t answer of RCT, journal source of SCI (Science Citation Index), manuscript length > 13 page and funding support.

**Conclusion:**

Since the publication of the PRISMA Statement, there has been an improvement in the quality of reporting of SRs and MAs on nursing interventions in patients with AD. More endorsement by journals of the report guideline for SRs/MAs may improve articles reporting quality, and the dissemination of reliable evidence to nurses. We recommend authors, readers, reviewers, and editors to become more acquainted with and to more strictly adhere to the PRISMA checklist.

## Background

Systematic reviews (SRs) and meta-analyses (MAs) keep up to date with developments in modern medicine. SRs and MAs can provide evidence of the value and feasibility on nursing interventions in patients with Alzheimer’s disease (AD), and to help clinicians, nurses, and policy makers to inform clinical decision-making. Despite their strengths, SRs have shown varying quality. As such, high quality of reporting is crucial to ensure reliable, transparent and accurate interpretation of evidence. Thus, some checklists have been published in an attempt to improve the reporting quality of systematic reviews and meta-analyses.

In an attempt to ensure validity of evidence and improve both the quality and completeness of reporting of SRs and MAs, a 27-item PRISMA statements have been published to assess the reporting quality of SRs in 2009, which was a successor to the original Quality Of Reporting Of Meta-analysis (QUOROM) guidelines [1, 2]. In recent years, a few of studies in various medical fields have been conducted for assessment of the qualities of SRs and MAs based on the fulfillments of PRISMA [3–5]. Cullis et al. [3] investigated that compliance with the PRISMA guidelines was poorer and the reporting quality of SRs and MAs in the published paediatric surgical literature needs to be improved. Panic et al. [4] found that the quality of reporting quality of SRs and MAs in journals in the field of gastroenterology and hepatology have significantly increased after PRISMA endorsement. Wasiak et al. [5] demonstrated that the reporting quality of SRs in burn care management is suboptimal and requires further improvement with stricter adherence by authors to the PRISMA checklist. Recently, more journals encourage the adoption of PRISMA include mandatory submission of reporting checklists and integration of PRISMA into the peer-review process [6]. Hence, an assessment of compliance before and after introduction of the PRISMA Statement will play an increasingly important role.

Alzheimer’s disease (AD) is a disease that causes significant functional impairments even in its early stage [7]. It is estimated that there will be 131.5 million people living with dementia globally by 2050. There are currently no treatments to reverse the course of AD, more by nursing intervention [8]. However, certain treatments, both pharmacological and psychotherapeutic, do achieve a slowing of the impairment of AD, especially regarding to psychological treatments [9]. So far, research on reporting quality evaluation of SRs and MAs on nursing interventions in patients with AD has not been available. Thus, this study evaluated the reporting quality of SRs and MAs on nursing interventions in patients with AD according to PRISMA checklists, and hoping to provide reference for authors, readers, reviewers, and journal editors. The secondary aim was to find that whether some predictive factors especially the publication of the PRISMA statement was associated with an improvement in reporting.

## Methods

### Search strategy

**Fig. 1:**
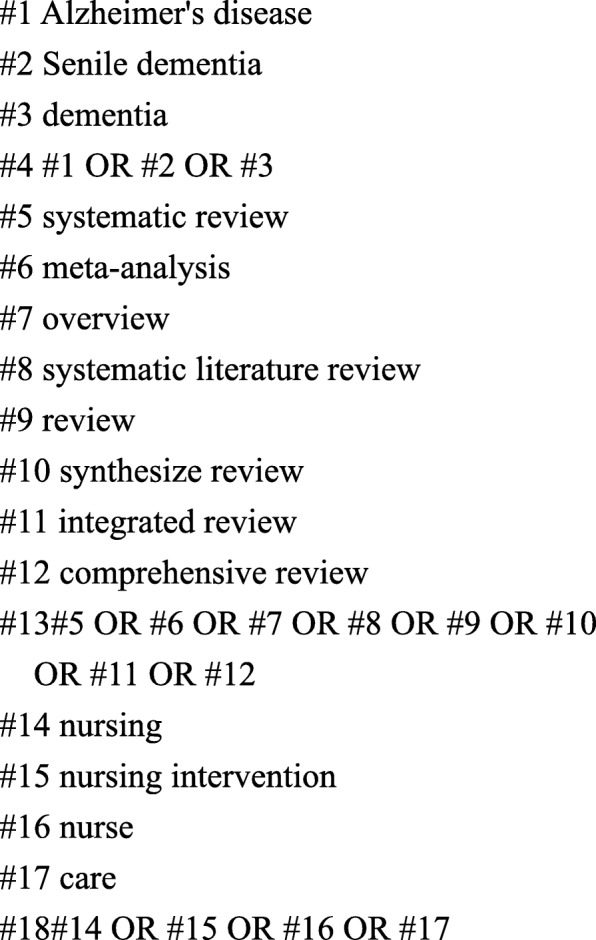
PubMed search strategy

The article that met inclusion criteria was identified by searching in PubMed, EMBASE and The Cochrane Library up to October 16th 2018. The language was limited to English. The search strategy included the use of Title/Abstract related to: (“Alzheimer’s disease” or “Senile dementia” or “dementia” or “AD”) and (“systematic review” or “meta-analyses” or “overview” or “systematic literature review” or “meta-analysis” or “review” or “synthesize review” or “integrated review” or “comprehensive review”) and (“nursing” or “nursing intervention” or “nurse” or “care”). Combined with Google Scholar and Baidu Scholar, we also scanned the reference lists manually reviewed from included articles to identify additional relevant studies. The search strategy for PubMed is outlined in Fig. 1.

### Study selection

To be eligible for inclusion, the reports have the following inclusion criteria: (1) articles being identified as SRs or MAs; (2) published in English language; (3) an article of nursing interventions in patients with Alzheimer’s disease. The exclusion criteria were as follows: (1) scoping review, traditional literature review and evidence-based commentaries; (2) an article of non-nursing interventions in patients with Alzheimer’s disease. Two investigators independently screened the titles and abstracts of all the retrieved articles using the inclusion criteria. Full-text articles were retrieved and reviewed independently in duplicate by two authors for potentially eligible articles. Any discrepancy was resolved by discussion or by asking a third reviewer if consensus could not be reached.

### Data extraction

Data extraction was performed independently on included articles by two investigators (S.X; L.H.H). Discrepancies and unobtainable data were resolved by group discussion between at least three investigators. General characteristics of SRs or MAs were extracted, including year of publication, author origin, the name and type of the journal, the authors’ affiliations, the number of affiliations, the article type, whether an RCT was identified, impact factor (IF), number of times cited, funding support, followed PRISMA guideline, international collaborative authorship, the number of included studies, topics of intervention, word count and protocol or registration, number of authors, journal source of SCI, manuscript length. Comparisons were made between manuscripts published before (2001–2009) and after (2011–2018) introduction of the PRISMA Statement to access the reporting quality. Since all three articles published in 2010 were submitted in 2009, we considered them as pre-published manuscripts.

### Quality assessment

According to the detailed per-item descriptions of the PRISMA Statement, appraisal of reporting was performed by two authors (S.X; L.H.H). The PRISMA statement covers seven modules with 27-items, ranging from 0 to 27. Each item was rated as yes, no or cannot answer, not applicable. A score of 1 was assigned to a “yes” answer, and a score of 0 was assigned to all other answers except “not applicable”. Items that were not applicable, such as those that only applied to MAs (ie, PRISMA items 14, 16 and 23), were scored 1. Therefore every study had an overall PRISMA score rated out of a maximum score of 27.

### Data analysis

Initially, descriptive analysis on the characteristics of the included SRs/MAs published before (2001–2009) and after (2011–2018) was conducted. Differences in categorical variables and comparison overall compliance in each PRISMA item were explored using the χ^2^ test (≤ 2009 vs. ≥2011). Comparisons of the PRISMA score between manuscripts published before (2001–2009) and after (2011–2018) were conducted using the t-test and covariance of analysis for adjusting confounding factors. A *p* <  0.05 was considered significant on statistical analyses. All the statistics analyses were conducted by SPSS 19.0 and RevMan software version 5.3.

## Result

### Search results

**Fig. 2:**
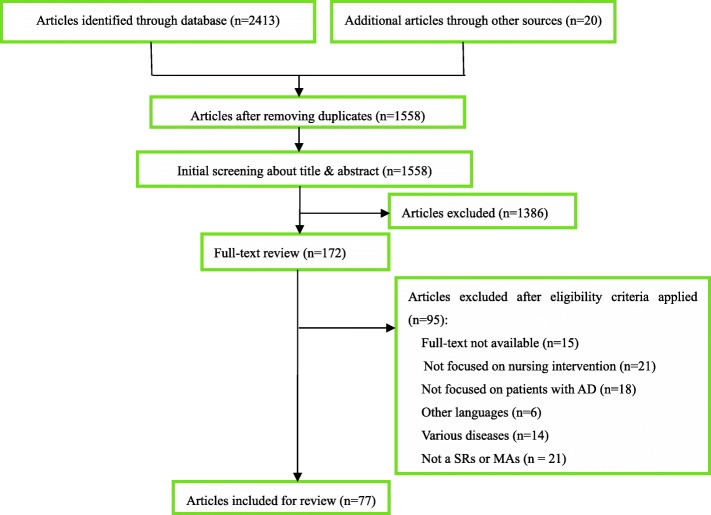
Flow chart of article screening and selection process

A total of 2413 articles were identified in initial search, whilst hand searching captured an additional 20 articles for potential inclusion. After removing duplicate articles, reviewing titles and abstracts, reviewing the full-text, there are a total of 77 articles to be included in this research finally. The process of literature retrieval was shown in a flow diagram (Fig. 2). The agreement between data extractors was moderate (kappa = 0.79, *P* <  0.01) for the PRISMA statement, which indicates a good level of agreement between scorers. All of the discrepancies were resolved by consensus between the two extractors.

### Descriptive characteristics

These articles were published in different journals from 2001 to 2018. Of included manuscripts, 18 (23.4%) were published before the PRISMA Statement (2001–2009) and 59 (76.6%) were published afterwards (2011–2018). There was higher score after publication of the PRISMA Statement than before (20.83 ± 3.78 vs 17.11 ± 4.56, *P* <  0.05). The characteristics of the 77 articles are shown in Table 1.

**Table 1:** Study characteristics before (2001–2009) and after (2011–2018) introduction of the PRISMA Statement

Characteristic	Before PRISMA (*n* = 18)	After PRISMA (*n* = 59)	Overall (*n* = 77)	χ^2^/ t	*P*
**Type of article**				4.53	0.09
Systematic reviews only	16 (88.9)	38 (69.6)	54 (70.1)		
Meta-analyses only	1 (5.6)	4 (6.8)	5 (6.5)		
Systematic reviews including meta-analyses	1 (5.6)	17 (28.8)	18 (23.4)		
**Origin region of first author**				1.69	0.68
Asia	2 (11.1)	12 (20.3)	14 (18.2)		
Europe	10 (55.6)	33 (55.9)	43 (55.8)		
U.S.A/Canada	3 (16.7)	9 (15.3)	12 (15.6)		
Australia	3 (16.7)	5 (8.5)	8 (10.4)		
**Number of authors**				4.91	**0.03**
1~ 4	15 (83.3)	32 (54.2)	47 (61.0)		
> 4	3 (16.7)	27 (45.8)	30 (39.0)		
**International collaborative authorship**				0.05	0.82
No	16 (88.9)	49 (83.1)	65 (84.4)		
Yes	2 (11.1)	10 (16.9)	12 (15.6)		
**Number of affiliations of first author**				1.96	0.16
1~ 3	15 (83.3)	39 (66.1)	54 (70.1)		
> 3	3 (16.7)	20 (33.9)	23 (29.9)		
**Affiliation of author**				1.36	0.61
Hospital	2 (11.1)	5 (8.5)	7 (9.1)		
University	12 (66.7)	46 (78.0)	58 (75.3)		
Institute	4 (22.2)	8 (13.6)	12 (15.6)		
**journal type of published article**				1.26	0.26
Nursing Journal	11 (61.1)	46 (78.0)	57 (74.0)		
Non Nursing Journal	7 (38.9)	13 (22.0)	20 (26.0)		
**Number of included studies**				0.05	0.83
< 10	5 (27.8)	18 (30.5)	23 (29.9)		
≥10	13 (72.2)	41 (69.5)	54 (70.1)		
**RCT identified**				10.44	**0.01**
Non-RCT	12 (66.7)	34 (57.6)	46 (59.7)		
Only RCT	1 (5.6)	21 (35.6)	22 (28.6)		
Unclear	5 (27.8)	4 (6.8)	9 (11.7)		
**Followed PRISMA guideline**				10.00	**< 0.01**
No	18 (100.0)	36 (61.0)	54 (70.1)		
Yes	0 (0.0)	23 (39.0)	23 (29.9)		
**Protocol or Registration**				0.73	0.39
No	15 (83.3)	41 (69.5)	56 (72.7)		
Yes	3 (16.7)	18 (30.5)	21 (27.3)		
**Journal source of SCI**				0.13	0.72
No	2 (11.1)	3 (5.1)	5 (6.5)		
Yes	16 (88.9)	56 (94.9)	72 (93.5)		
**Manuscript length (no. of pages)**				0.53	0.47
1~ 13	8 (44.4)	32 (54.2)	40 (51.9)		
> 13	10 (55.6)	27 (45.8)	37 (48.1)		
**Funding support**				0.07	0.79
No	11 (61.1)	34 (57.6)	45 (58.4)		
Yes	7 (38.9)	25 (42.4)	32 (41.6)		
**The total score** ($\overline{x}$± s)	17.11 ± 4.59	20.83 ± 3.78	19.96 ± 4.26	−3.47	**< 0.01**

Asia (China, Hong kong, Taiwan, Jepan, Korea, Singapore); Europe (Britain, Netherlands, Germany, Sweden, Spain, Norway, Finland, Belgium, Ireland)

Significant results are shown in bold

### Reporting quality of included reviews

**Fig. 3:**
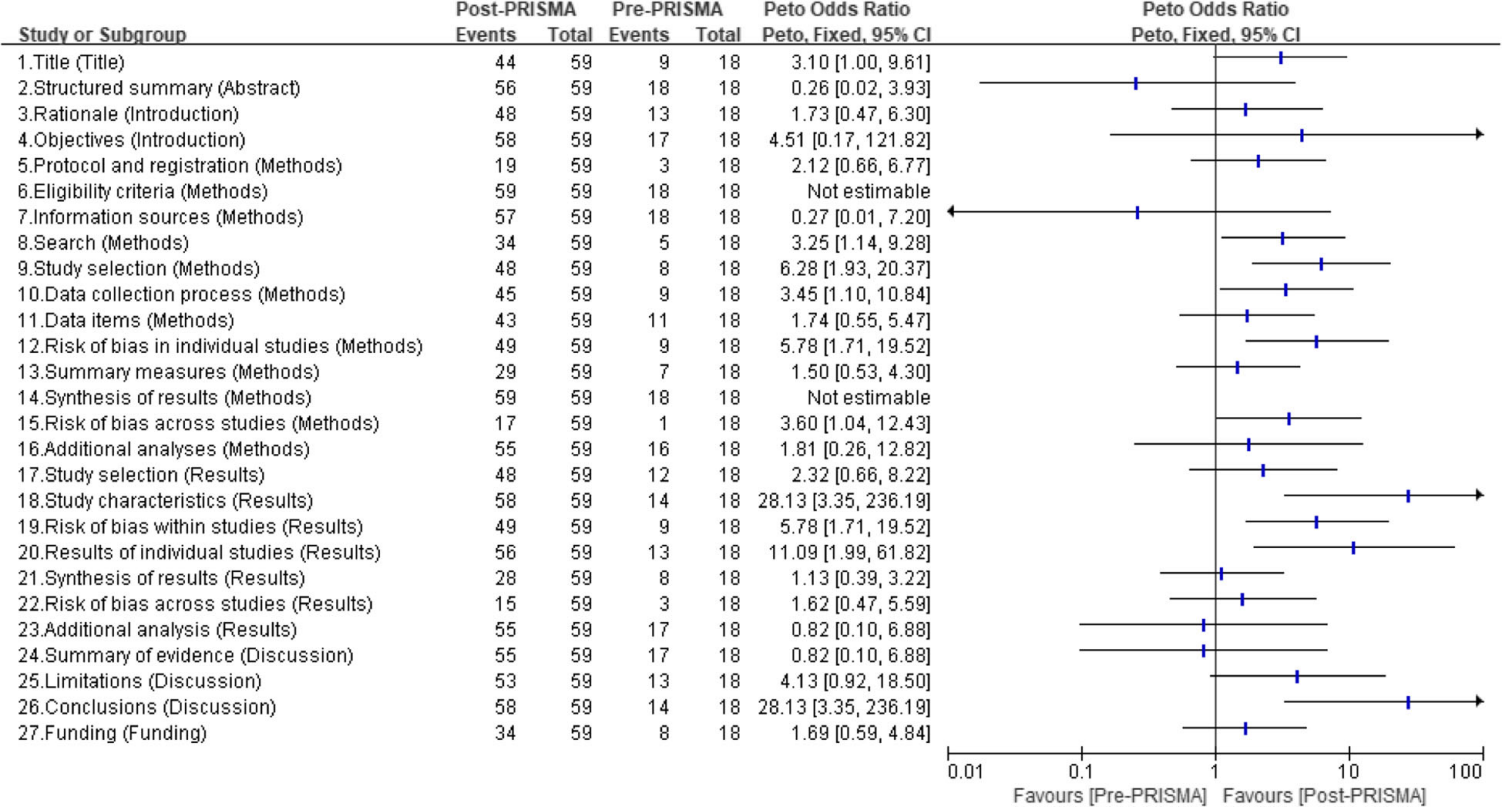
Comparison of pre-PRISMA and post-PRISMA periods for each PRISMA item

To determine whether the publication of the PRISMA statement was associated with an improvement in the quality of reporting, the period ≤2009 was compared with ≥2011 in each of the PRISMA criteria (Fig. 3). The results showed that there was an improvement in the following items after the PRISMA statement was released, which was significant difference: title (item 1, 50.0% vs 74.6%,OR = 3.10, 95CI%:1.00–9.61), search (item 8, 27.8% vs 57.6%,OR = 3.25, 95CI%:1.14–9.28), study selection (item 9, 44.4% vs 81.4%, OR = 6.28, 95CI%:1.93–20.37),Data collection process (item 10, 50.0% vs 76.3%, OR = 3.45, 95CI%:1.10–10.84), risk of bias in individual studies (item 12, 50.0% vs 83.1%,OR = 5.78, 95CI%:1.71–19.52), risk of bias across studies (item 15, 5.6% vs 28.8%, OR = 3.60, 95CI%:1.04–12.43), study characteristics (item 18, 77.8% vs 98.3%, OR = 28.13, 95CI%:3.35-236.19), risk of bias with studies (item 19, 50.0% vs 83.1%,OR = 5.78, 95CI%: 1.71-19.52), results in individual studies (item 20, 72.2% vs 94.9%, OR = 11.09, 95CI%:1.99–61.82), conclusions (item 26, 77.8% vs 98.3%, OR = 28.13, 95CI%:3.35–236.19). Prevalence of reporting of these items was 3.10 to 28.13 times higher than before PRISMA Statement introduction. However, other items didn’t show significant difference or there was no improvement after the PRISMA statement was released. Per-item PRISMA analysis reveals six items that have less than 50% adherent studies (item 5, protocol and registration; item 13, summary measures; item 15, risk of bias across studies; item 21 and 22, synthesis of results and risk of bias across studies), while others have high compliance before and after PRISMA publication.

### Univariable and covariance analysis on publication time

Univariable and covariance analysis of study demographics using PRISMA score as dependent variables identified several significant trends showed in Table 2. The following factors were significant in univariate analysis concerning PRISMA score: after PRISMA introduction (Mean difference (MD) = 3.72), SRs including MAs (MD = 5.00), number of authors > 4 (MD = 3.83), number of affiliations of author > 3 (MD = 2.47), affiliation of author is institute (MD = − 2.45), journal type is non nursing journal (MD = 2.58), only RCT (MD = 3.19), followed PRISMA guideline (MD = 2.91), having protocol or registration (MD = 5.03), journal source of SCI (MD = 7.44), manuscript length > 13 (MD = 2.68), funding support (MD = 3.49). In covariance analysis, those variables that were significant in univariate analysis were controlled to access reporting quality published before (2001–2009) and after (2011–2018). The result showed that PRISMA score improved after introduction of the PRISMA Statement (MD = 1.65). After controlling for the confounding factors, there were higher PRISMA score for systematic reviews including meta-analyses (increased by 2.57), protocol or registration (increased by 2.75), can't answer of RCT (reduced to 2.19), journal source of SCI (increased by 3.76), manuscript length > 13 page (increased by 1.63) and funding support (increased by 2.41).

**Table 2:** Univariable and covariance analysis for predictive factors on PRISMA scores

			PRISMA univariable analysis			PRISMA covariance analysis	
Variable	Category/unit	Mean Diff.	95% CI	*P* value	Mean Diff.	95% CI	*P* value
**Publication time**	Before PRISMA	Reference			Reference		
	After PRISMA	3.72	1.59, 5.85	**< 0.01**	1.65	0.19, 3.10	**0.03**
**Type of article**	Systematic reviews only	Reference			Reference		
	Meta-analyses only	1.93	−1.55, 5.42	0.19	1.71	−0.78, 4.20	0.18
	Systematic reviews including meta-analyses	5.00	2.97, 7.03	**< 0.01**	2.57	1.02, 4.13	**< 0.01**
**Origin region of first author**	Asia	Reference			Reference		
	Europe	−0.23	- 2.82, 2.37	0.86	0.93	−0.91, 2.76	0.32
	U.S.A/Canada	−2.55	− 5.86, 0.76	0.13	0.67	− 1.48,1.97	0.76
	Australia	− 2.21	−5.94, 1.52	0.24	0.07	−2.70, 2.57	0.96
**Number of authors**	1~ 4	Reference			Reference		
	> 4	3.83	2.04, 5.62	**< 0.01**	1.15	−0.33, 2.53	0.13
**International collaborative authorship**	No	Reference			Reference		
	Yes	0.85	−1.84, 3.51	0.54	−1.16	− 2.80, 0.47	0.16
**Number of affiliations of author**	1~ 3	Reference			Reference		
	> 3	2.47	0.43, 4.52	**0.02**	−0.18	−1.59, 1.22	0.79
**Affiliation of first author**	University	Reference			Reference		
	Hospital	−1.16	− 4.52, 2.20	0.49	− 0.86	−1.19, 2.92	0.40
	Institute	−2.45	- 5.11, 0.21	**0.07**	0.38	−1.33, 2.09	0.66
**journal type of published article**	Nursing Journal	Reference			Reference		
	Non Nursing Journal	2.58	0.44, 4.72	**0.02**	0.30	−1.14, 1.73	0.68
**Number of included studies**	< 10	Reference			Reference		
	≥10	−0.74	− 2.186, 1.38	0.49	− 1.06	−2.32, 0.19	0.10
**RCT identified**	Non-RCT	Reference			Reference		
	Only RCT	3.19	1.31, 5.06	**< 0.01**	−0.16	−1.73, 1.41	0.84
	Can’t answer	−4.59	−7.22, − 1.95	**< 0.01**	−2.19	−4.20, − 0.18	**0.03**
**Followed PRSIMA guideline**	No	Reference			Reference		
	Yes	2.91	0.89, 4.93	**< 0.01**	1.22	−0.18 2.63	0.09
**Protocol or Registration**	No	Reference			Reference		
	Yes	5.03	3.18, 6.88	**< 0.01**	2.75	1.38, 4.12	**< 0.01**
**Journal source of SCI**	No	Reference			Reference		
	Yes	7.44	3.89, 11.00	**< 0.01**	3.76	1.34, 6.18	**< 0.01**
**Manuscript length (no. of pages)**	1~ 13	Reference			Reference		
	> 13	2.68	0.83, 4.52	**< 0.01**	1.63	0.42, 2.84	**< 0.01**
**Funding support**	No	Reference			Reference		
	Yes	3.49	1.69, 5.29	**< 0.01**	2.41	1.22, 3.60	**< 0.01**

Significant results are shown in bold

## Discussion

The number of SRs/MAs about AD has been accumulating at an increasing rate in recent years. Although the quality of the reporting was suboptimal, the reporting quality of SRs and MAs of nursing interventions in patients with AD had been an improvement in the quality of reporting after the PRISMA statement was released. On average, PRISMA score with before and after publication of the PRISMA Statement was 17.11 and 20.83, respectively. Similarly, there was significant difference in compliance with some items of the PRISMA Statement before and after its introduction.

The articles [4, 10, 11] have evaluated compliance with the PRISMA Statement in other medical disciplines. Tunis et al. focused on SRs and MAs published from 2007 to 2011 in 11 high-impact radiology journals and showed an increase from an average of 20.90 of 27 items reported prior to PRISMA publication to an average of 22.60 of 27 items reported after publication of PRISMA [10]. An assessment SRs and MAs of diagnostic tests published by in China revealed that there had been some statistically significant improvement in total compliance for 9 PRISMA items after the PRISMA was released [11]. Like our study, the study demonstrated that several potential predictive factors for superior quality of urological meta-analyses were identified, including funding support, following PRISMA guideline, and “a priori” design [12]. There are discrepancies in evaluation quality of SRs among various medical specialties.

The main strengths of this study include the focused search and selection of SRs/MAs, comprehensive assessment, and planned linear regression analyses. For selection of SRs/MAs, we not only included SRs but also MAs because our study has to be more comprehensive and objective. In addition, some of the assessment items only applied to meta-analyses, such as PRISMA items 14, 16, 23. To minimize bias against systematic review, we identified these items as not applicable and considered the item to be fulfilled when the total number of completed items was compiled. Thus, they have a more consistent score criterion. Since the PRISMA statement was published in 2009, we did not use this guidance to assess the quality of SRs/MAs published in 2010, which was sufficient to allow adequate uptake by authors and editors before assessment. Also, our results would have more credibility and our conclusions would have a more specific implication.

It is worth noting that a number of PRISMA items (item 1, item 8, item 9, item 10, item 12, item 15, item 18, item 19, item 20 and item 26) have increased in reporting after PRISMA Statement introduction. Prevalence of reporting of these items has gone up 3.10 to 28.13 times after PRISMA Statement introduction. The majority of included studies were described as SRs and/or MAs within the title (item 1). In order to identify instantly by the reader and to improve indexing, the title of the paper should include the PICOS approach (participants, intervention, comparison, outcome and study design), which will make readers know potential high level of evidence and help them decide whether the paper is worth reference [13]. The study selection processes (item 9) perform that authors identify records from their search and sequentially exclude records according to eligibility criteria. It protects the rigor and science of the SRs and/or MAs. Of greater importance was the high rate of reporting of study characteristics (item 18). For readers to gauge the validity and applicability of a SR’s results, authors need to report information (such as PICOS, questions related to specific patients, interventions, comparisons,) about the included studies [14].

It is generally believed that PRISMA statement is significant for authors of SRs/MAs to follow to improve their reporting quality and transparency. But the poorly reported items need to further improvement. Whether it is before or after publication, five items with compliance less than 50% include items related to describing the review protocol and registration, describing the risk of bias, describing summary measures, and describing synthesis of results (items 5, 15, 22, 13, and 21, respectively). Based on our data, the generally lower compliance was reported and reflected a lack of awareness regarding protocols and registration (items 5) and risk of bias across studies (items 15 and 22) among authors as to their importance and potential implications. The use of protocol and review registration is important because a protocol makes more accurate for authors’ researches and supports better transparency during the review process. As for SRs, only Cochrane reviews require the authors to publish a peer-reviewed protocol before conducting the review. Previous studies have shown that Cochrane reviews appear to have higher reporting quality than SRs or MAs published in paper-based journals [3, 5, 15]. The registration resources such as PROSPERO [16] and Systematic Reviews [17] can make authors easily use to obtain information regarding protocol and registration. Risk of bias across studies is typically performed by testing for publication bias [18]. Publication and outcome reporting biases are common in the literature and are likely to affect summary effect estimate [19–21]. To explore the publication biases, funnel plots and tests for funnel plot asymmetry should be provided in meta-analyses with more than 10 included studies [22]. Therefore, authors should carefully consider whether their results are affected by these common biases by the examination of funnel plots or the performance of statistical tests [23].

Covariance analysis was conducted to explore characteristics associated with overall reporting quality. Overall, SRs including MAs score higher with regards PRISMA scores, than SRs alone. Compared with the traditional descriptive review, SRs including MAs can make validity and reliability of evidences. The reporting quality of SCI articles was better compared with non-SCI articles. For more than 50 years, the SCI database has been continuously developed and has become the most important large-scale database in the contemporary world. It is one of the important indicators to evaluate the academic level of a country, a scientific research institution, an institution of higher learning, a journal, and even a researcher. Manuscript length was also associated with higher compliance with the PRISMA statements. Manuscript length limits imposed by some journals are one of the major obstacles and these findings showed that reporting quality is compromised when inadequate space is provided in all sections [24]. We also found evidence that funding support with better reporting quality were more likely to call for further research. The impact of funding on author conclusions has long been recognized in randomized trials and MAs of clinical trials [25, 26]. It is necessary to improve awareness among journal reviewers, editors, funders, institutions, and readers. We call for journals to make the PRISMA checklist mandatory for the electronic submission of SRs.

There are some limitations to this study. Firstly, this study was restricted to SRs/MAs examining nursing interventions in Alzheimer’s disease only, thus excluding a large number of reviews that may have produced a different response. Secondly, we were restricted to electronic databases, which may not have been representative of all indexed studies. And only articles written in English were enrolled for analysis.

## Conclusion

Overall, some improvements in compliance with some items of the PRISMA Statement were found SRs and MAs articles of nursing interventions in patients with Alzheimer’s disease after the publication of the PRISMA statement, even the overall quality of reporting. After controlling for the confounding factors, there were higher PRISMA score for systematic reviews including meta-analyses, can’t answer of RCT, protocol or registration, can't answer of RCT, journal source of SCI, manuscript length > 13 page and funding support. But clinicians, nurses and investigators should critically appraise all reports of SRs/MAs before considering the results. More endorsement by journals of the report guideline for SRs/MAs may improve articles quality, and the dissemination of reliable evidence to nurses. We recommend readers, reviewers, and editors to become more acquainted with to the PRISMA checklist. In addition, authors should stricter adhere to the PRISMA checklist when conducting a SR/MA.

## Abbreviations

AD: Alzheimer’s disease
IF: Impact factor
MAs: Meta-analyses
PRISMA: Preferred Reporting Items for Systematic Reviews and Meta Analyses
SRs: Systematic reviews
